# Multiple Roles of the Extracellular Vestibule Amino Acid Residues in the Function of the Rat P2X4 Receptor

**DOI:** 10.1371/journal.pone.0059411

**Published:** 2013-03-21

**Authors:** Milos B. Rokic, Stanko S. Stojilkovic, Vojtech Vavra, Pavlo Kuzyk, Vendula Tvrdonova, Hana Zemkova

**Affiliations:** 1 Department of Cellular and Molecular Neuroendocrinology, Institute of Physiology of the Academy of Sciences of the Czech Republic, Prague, Czech Republic; 2 Department of Animal Physiology, Faculty of Science, Charles University, Prague, Czech Republic; 3 Section on Cellular Signaling, Program in Developmental Neuroscience, National Institute of Child Health and Human Development, National Institutes of Health, Bethesda, Maryland, United States of America; Universidade Federal do ABC, Brazil

## Abstract

The binding of ATP to trimeric P2X receptors (P2XR) causes an enlargement of the receptor extracellular vestibule, leading to opening of the cation-selective transmembrane pore, but specific roles of vestibule amino acid residues in receptor activation have not been evaluated systematically. In this study, alanine or cysteine scanning mutagenesis of V47–V61 and F324–N338 sequences of rat P2X4R revealed that V49, Y54, Q55, F324, and G325 mutants were poorly responsive to ATP and trafficking was only affected by the V49 mutation. The Y54F and Y54W mutations, but not the Y54L mutation, rescued receptor function, suggesting that an aromatic residue is important at this position. Furthermore, the Y54A and Y54C receptor function was partially rescued by ivermectin, a positive allosteric modulator of P2X4R, suggesting a rightward shift in the potency of ATP to activate P2X4R. The Q55T, Q55N, Q55E, and Q55K mutations resulted in non-responsive receptors and only the Q55E mutant was ivermectin-sensitive. The F324L, F324Y, and F324W mutations also rescued receptor function partially or completely, ivermectin action on channel gating was preserved in all mutants, and changes in ATP responsiveness correlated with the hydrophobicity and side chain volume of the substituent. The G325P mutant had a normal response to ATP, suggesting that G325 is a flexible hinge. A topological analysis revealed that the G325 and F324 residues disrupt a β-sheet upon ATP binding. These results indicate multiple roles of the extracellular vestibule amino acid residues in the P2X4R function: the V49 residue is important for receptor trafficking to plasma membrane, the Y54 and Q55 residues play a critical role in channel gating and the F324 and G325 residues are critical for vestibule widening.

## Introduction

The purinergic P2X receptors (P2XRs) are ATP-gated cation channels that are expressed in a wide range of species from amoeba to humans [1]. These receptors have a widespread distribution in neuronal and non-neuronal cells and are involved in many physiological and pathophysiological processes, including pain perception, modulation of neurotransmission, neuron–glia communication, hormone release, contraction of smooth muscles, apoptosis, cell proliferation, aggregation of thrombocytes, inflammation and cancer [2]–[5]. The P2X4R subtype is expressed in highest concentration throughout the central and peripheral nervous system, and its activation facilitates Ca^2+^ influx [6] and neurotransmitter release [7]. The role of P2X4Rs was also confirmed in tactile allodynia, the hypersensitivity of nociceptive transmission in injured primary sensory neurons [8] and in the learning and memory processes [9]. Another potentially important characteristic of the brain-localized P2X4Rs is their sensitivity to ethanol P2X4Rs [10].

In mammals, seven P2X subunits (termed P2X1–7) have been found [11] and the P2XRs are organized as trimeric homomers or heteromers [12], [13]. Each of the receptor subunits has two transmembrane domains (TM1 and TM2), with intracellular N- and C-termini and a large extracellular loop [11]. P2XRs have three intersubunit ATP-binding sites [14] and 2–3 ATP molecules are required for their activation [15], [16]. The homomeric P2X4R is rapidly activated upon ATP binding, desensitizes at a moderate rate, and has an inwardly rectifying current voltage relationship that reverses at 0 mV [17]. The receptor is relatively resistant to the P2XR antagonists suramin and PPADS [18] and is allosterically regulated by ivermectin (IVM) [19], [20]. When applied extracellularly, IVM increases the sensitivity of the receptors to ATP, enhances the maximum current amplitudes (I_max_) and significantly prolongs the deactivation kinetics [21], [19], [22]. Activated P2X4R is rapidly endocytosed and trafficked to lysosomes [23]–[25].

The crystal structures of the P2X4R from zebrafish (zfP2X4R) confirmed the trimeric architecture of this receptor and the α-helical organization of the TMs, and showed the existence of three ectodomain vestibules [26], [27]. Several recent functional studies on P2X2R and P2X4R, in combination with mutagenesis, have addressed the role of lateral fenestrations of extracellular vestibule in receptor function using cysteine mutants and modification of introduced residue by methanethiosulfonate reagents, cadmium ion accessibility or artificial disulphide bonding disruption with dithiothreitol. The main focus in these studies was on the E51 residue [28], the E56, D58 and T57 residues that line the lateral portals of closed human P2X4R in their narrowest region [29], the I333 to L358 TM2 residues of P2X2R [30], and E51–D58 and K326–N338 residues of P2X2R [31] (P2X4 numbering). These studies indicated that ions access and enter the pore through lateral fenestrations of extracellular vestibule and that negatively charged side chains of portals are involved in ion selectivity, but did not clarify the specific role of amino acid residues in this region on receptor function.

Furthermore, cysteine-scanning mutagenesis of P2X1R residues E52–G96, showed that, apart from K68 and K70 residues that are important for ATP binding, all vestibule mutants are functional [32], which is not a case of P2X2R [33], [31]. Analysis of alanine-substituted mutants of extracellular peptide segment proximal to TM2 also showed that conserved polar amino acids in this region of P2X1R are not essential for ATP binding [34], whereas conserved residues within the G316–I333 region of the rat P2X4R (rP2X4R) are important for signal transduction between the ATP binding domain and the channel gate in the TM2 [35].

Here we summarize our work on the structural and functional analysis of the rP2X4R extracellular vestibule. Initially, we performed alanine- and cysteine-scanning mutagenesis of amino acid residues V47 to V61 and F324 to N338 to identify residues that are potentially important for the receptor’s function. We used both types of mutants because to be able to compare our results to the limited work performed previously. Scanning mutagenesis helped us to identify five residues, V49, Y54, Q55, F324, and G325, as poorly responsive to ATP. We then used a series of additional mutants of these residues, and the wild type (WT) and all mutants were also probed with IVM. These experiments, supplemented with correlations studies and structural analysis, indicated multiple roles of the extracellular vestibule amino acid residues in the function of P2X4R.

## Methods

### Cells Culture and Transfection

To express the recombinant channels, we used human embryonic kidney (HEK) 293T cells (American Type Culture Collection, Rockville, MD, USA) grown in Dulbecco modified Eagle’s medium supplemented with 10% fetal bovine serum, 50 U/ml penicillin and 50 µg/ml streptomycin in a humidified 5% CO_2_ atmosphere at 37°C. Transfection was done using 2 µg of either WT or mutant receptor DNA with 2 µl of jetPRIME™ reagent in 2 ml of Dulbecco modified Eagle’s medium, according to manufacturer’s instructions (PolyPlus-transfection, Illkirch, France).

### DNA Constructs

cDNAs encoding the sequences of rP2X4R and mutated subunits were subcloned into the pIRES2-EGFP vector (Clontech, Mountain View, CA, USA). To generate the mutants, oligonucleotides (synthesized by VBC-Genomics, Vienna, Austria and Sigma Chemical Company, USA) containing specific point mutations were introduced into the rP2X4/pIRES2-EGFP template using PfU Ultra DNA polymerase (Fermentas international Inc, USA). A High-Speed Plasmid Mini Kit (Geneaid, Shijr City, Taipei County, Taiwan) was used to isolate the plasmids for transfection. Dye terminator cycle sequencing (ABI PRISM 3100, Applied Biosystems, Foster City, CA) was used to identify and verify the presence of the mutations. The sequencing was performed by the DNA Sequencing Laboratory, Institute of Microbiology, ASCR, Prague.

### Patch Clamp Recordings

ATP-induced currents were recorded from whole cells clamped to −60 mV using an Axopatch 200B patch-clamp amplifier (Axon Instruments, Union City, CA). The recordings were captured and stored using the Digidata 1322A and pClamp9 software package. During the experiments, the cell culture was perfused with an extracellular solution containing: 142 mM NaCl, 3 mM KCl, 2 mM CaCl2, 1 mM MgCl2, 10 mM HEPES and 10 mM D-glucose, adjusted to pH 7.3 with 1 M NaOH. The patch electrodes were filled with a solution containing: 154 mM CsCl, 11 mM EGTA and 10 mM HEPES, adjusted to pH 7.2 with 1.6 M CsOH. Unless otherwise stated, ATP was applied for 2–10 s at different concentrations to evoke inward currents and one or two responses were recorded from one cell to prevent desensitization of the receptors. To estimate the concentration producing 50% of the maximal response (EC_50_), the responses from different cells were pooled. Consequently, the EC_50_ ATP concentrations in naïve cells found here (2.3±0.4 µM) was about two times lower as compared to value obtained by others (5.2±1 µM) [36] and comparable to that obtained by others using the same method (3.1±0.5) [35]. Data points are presented as mean ± SEM from 5–35 cells. In some experiments, whole cell currents were measured in the presence of 3 µM IVM after preincubation for several minutes. The control and ATP-containing solutions were applied via a rapid (exchange time ∼30–40 ms) superfusion system (RSC-200, BIOLOGIC, Claix, France).

### Quantification of Receptor Expression

To determine the amount of rP2X4R mutants that were membrane-bound, all membrane proteins were labeled by biotin using the Pierce® Cell Surface Protein Isolation Kit, according to the manufacturer’s instructions (Thermo Scientific, Rockford, USA). Total protein samples were collected after cell lysis and centrifugation of cell debris. The membrane protein fraction was isolated from cell lysate by affinity column binding to avidin, followed by washing and elution in SDS sample buffer. Both fractions were subjected to SDS-PAGE, transferred to PVDF, immunoblotted with an anti-rP2X4R monoclonal antibody (Alomone Labs, Ltd. Israel) and detected using horseradish peroxidase coupled to anti-rabbit Fc-IgG antibody in a luminol assay. The luminescent signals were captured using a Luminescent Image Analyzer LAS-1000plus (Fuji Photo Film Co., Ltd. Japan).

### Calculations and Molecular Model Representations

The concentration-response data points were fitted with the equation *y = I_max_/[1+ (EC_50_/x)^h^*], where *y* is the amplitude of the current evoked by ATP, *I_max_* is the maximum current amplitude induced by 100 µM ATP, EC_50_ is the agonist concentration producing 50% of the maximal response, *h* is the Hill coefficient, and *x* is the concentration of ATP (SigmaPlot 2000 v9.01; SPSS Inc., Chicago, IL). Hill coefficient was fixed to 1.3 in all experiments, a value obtained for the WT receptor by fitting. All numerical values in the text are reported as the mean ± SEM. Significant differences (**p<0.01 and *p<0.05) between means were determined using SigmaStat 2000 v9.01. The data for alanine and cysteine mutants were combined and analyzed by ANOVA and Tukey’s post hoc test. All graphical representations of the protein structure were prepared using PyMOL software (DeLano Scientific LLC, USA), and the models were extracted from the Brookhaven Protein Data Bank under accession number 4DW1 for the zfP2X4R in the ATP-bound open state and 4DW0 for receptor in the apo-closed state.

## Results

### Identification of Residues Critical for Receptor Function

**Figure 1:**
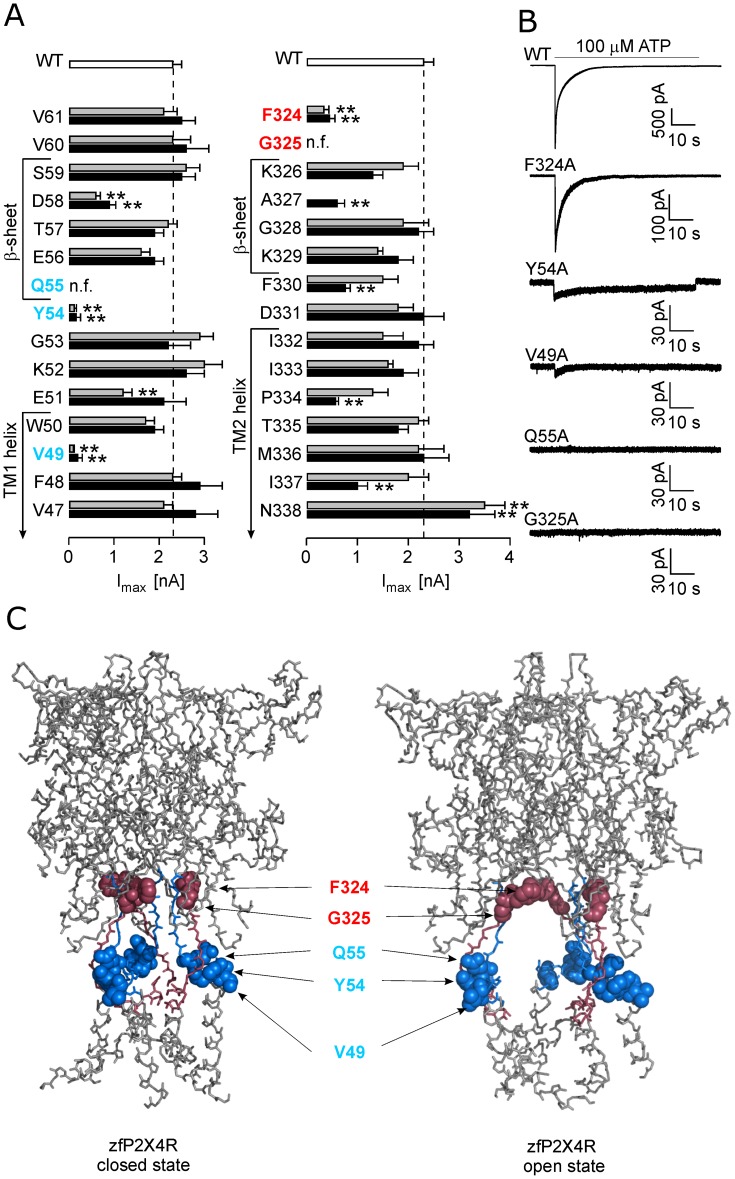
Effect of alanine and cysteine point mutations on the maximum current amplitude. (A) Alanine and cysteine scanning mutagenesis of residues V47–V61 and F324–N338 that contain the upper parts of the TM1 and TM2 helices and the β-sheets in the open state. The maximum amplitude of currents (I_max_) induced by 100 µM ATP in the wild type (WT; white bars) and cysteine (dark bars) and alanine (gray bars) mutant receptors. The receptors most affected had mutations at positions V49, Y54, Q55 (blue), F324 and G325 (red). The data are expressed as the mean ± SEM from 10–96 cells; **p<0.01 between WT and alanine or cysteine mutants. (B) The pattern of the ATP-induced currents by the WT and low-responsive rP2X4R alanine mutants. The horizontal bars indicate the duration of the application of 100 µM ATP (60 s). Notice the variable Y-scales for the WT and mutant receptors. (C) The topology of low-active residue mutants in the zfP2X4R in the apo-closed state (left) and ATP-bound open state (right); the mutated regions containing the upper parts of the TM1 and TM2 are shown in blue and red, respectively; affected mutated residues (rP2X4 numbering) are shown in red and blue spheres. Identity between rat and zebrafish P2X4R in amino acid sequences V47–V61 and F324–N338 is 67% and all functionally important residues are identical in both receptors.

To study the functional importance of the amino acid residues of the extracellular vestibule and lateral fenestrations of the rP2X4R, we performed scanning mutagenesis of amino acid residues V47–V61 and F324–N338. Both alanine- and cysteine-scanning mutagenesis were performed to provide an adequate comparison to the work performed previously. WT and mutant receptors were expressed in HEK293T cells, and ATP-induced currents were measured 24–48 h after transfection using the whole-cell patch clamp method. The results are summarized in Fig. 1 and Tables 1 and 2 as the mean ± SEM values for EC_50_ and I_max_ evoked by 100 µM ATP.

**Table 1:** Alanine- and cysteine-scanning mutagenesis of the V47–V61 rP2X4R segment.

P2X_4_R	EC_50_	I_max_
	[µM]	[nA]
WT	2.3±0.4	2.3±0.2
V47A	5.2±0.7*	2.1±0.2
V47C	2.3±0.7	2.8±0.5
F48A	2.4±0.3	2.3±0.2
F48C	2.0±0.9	2.9±0.5
V49A	n.d.	0.04±0.01**
V49C	n.d.	0.21±0.13**
V49D	1.6±0.4	1.7±0.3
V49W	2.7±0.4	2.3±0.3
V49L	1.6±0.4	3.1±0.4
W50A	3.6±0.3	1.9±0.2
W50C	4.4±0.8	2.2±0.4
E51A	1.6±0.6	1.2±0.2**
E51C	3.5±0.5	2.1±0.5
K52A	3.1±1.1	3.0±0.4
K52C	2.4±0.6	2.6±0.4
G53A	2.8±0.6	2.9±0.3
G53C	3.8±0.5	2.2±0.5
Y54A	n.d.	0.12±0.06**
Y54C	n.d.	0.16±0.10**
Y54W	6.3±2.6	1.6±0.2
Y54F	2.7±0.5	2.4±0.5
Y54L	n.f.	<0.1**
Q55A	n.f.	<0.1**
Q55C	n.f.	<0.1**
Q55T	n.f.	<0.1**
Q55N	n.f.	<0.1**
Q55E	n.d.	0.16±0.05**
Q55K	n.f.	<0.1**
E56A	2.0±0.9	1.6±0.2
E56C	3.9±0.7	1.9±0.2
T57A	1.9±0.6	2.2±0.3
T57C	2.0±0.3	1.9±0.2
D58A	3.2±1.3	0.6±0.1**
D58C	2.3±0.9	0.9±0.1**
S59A	2.9±1.3	2.6±0.4
S59C	2.0±0.5	2.5±0.3
V60A	2.4±0.6	2.3±0.4
V60C	2.1±0.8	2.6±0.5
V61A	2.9±0.4	2.1±0.3
V61C	3.7±1.5	2.5±0.3

In this and Table 2, the potency is expressed as the ATP concentration producing 50% of the maximal response (EC_50_), and the efficacy as the maximum induced current (I_max_) in response to 100 µM ATP. The I_max_ data are expressed as the mean ± SEM from 12–40 measurements per mutant and 96 measurements from the wild type (WT) receptor. n.d., not determined; n.f., nonfunctional;

** p<0.01, between mutant and WT (used for I_max_ measurements);

* p<0.05 between mutant and WT (used for EC_50_ estimation).

**Table 2:** Alanine- and cysteine-scanning mutagenesis of the F324–N338 rP2X4R segement.

P2X_4_R	EC_50_	I_max_
	[µM]	[nA]
WT	2.3±0.4	2.3±0.2
F324A	1.1±0.8	0.34±0.10**
F324C	0.6±0.9	0.27±0.05**
F324L	2.9±1.1	0.9±0.1**
F324Y	4.5±1.7	0.8±0.1**
F324W	8.9±2.7	2.7±0.5
G325A	n.f	<0.1**
G325C	n.f	<0.1**
G325P	3.0±1.3	1.7±0.2
K326A	1.7±0.6	1.9±0.3
K326C	2.4±0.6	1.3±0.2
A327C	1.7±0.6	0.6±0.2**
G328A	2.6±0.9	1.9±0.5
G328C	2.7±0.6	2.2±0.3
K329A	4.9±1.5	1.4±0.1
K329C	4.0±1.2	1.8±0.3
F330A	3.2±1.2	1.5±0.3
F330C	4.0±1.2	0.8±0.1**
D331A	1.5±0.2	1.8±0.3
D331C	2.1±0.2	2.3±0.4
I332A	1.3±0.3	1.5±0.4
I332C	1.6±0.3	2.2±0.3
I333A	2.9±0.8	1.6±0.1
I333C	2.8±1.1	1.9±0.3
P334A	1.0±0.3*	1.3±0.3
P334C	1.4±0.4	0.6±0.06**
T335A	1.8±0.6	2.2±0.2
T335C	2.6±0.5	1.8±0.2
M336A	2.5±0.3	2.2±0.5
M336C	1.2±0.2	2.3±0.5
I337A	2.9±1.3	2.0±0.4
I337C	4.1±0.6	1.0±0.2**
N338A	2.0±0.2	3.5±0.4**
N338C	1.1±0.2	3.2±0.5**

The I_max_ data shown are expressed as the mean ± SEM from 10–38 measurement per mutant.

** p<0.01, between mutant and WT (used for I_max_ measurements);

* p<0.05 between mutant and WT (used for EC_50_ estimation).

Compared to the cells expressing WT receptors, the EC_50_ and I_max_ values were not significantly different for the following alanine and cysteine mutants: F48, W50, K52, G53, E56, T57, S59, V60, V61, K326, G328, D331, I332, I333, T335, and M336. Following mutants exhibited significant (p<0.01 between WT and alanine or cysteine mutants) changes in the I_max_ values: E51A, D58A, D58C, A327C, F330C, P334C, I337C, N338A and N338C (Fig. 1A). The V47A and P334A mutants also exhibited significant (p<0.05) change in the EC_50_ value for ATP compared to WT receptor (Table 1). Because the I_max_ values for the I337A, P334A, F330A, and E51C mutants and the EC_50_ value for the V47C and P334C mutants were comparable to WT receptor, and E51A, D58A, D58C, A327C, F330C, and I337C mutants were characterized previously [37]–[40] [31], [30], [29], [28], these mutants were not studied further.

Substitution of five residues (V49, Y54, Q55, F324 and G325) with both alanine and cysteine resulted in low functional or non-functional receptors, that responded with I_max_ amplitudes less than 20% of that observed in the WT receptor (Fig. 1A) and their EC_50_s could not be determined, except for F324 mutants (Table 2). Figure 1B shows the typical current responses of the alanine mutants to 100 µM ATP for 60 s and Fig. 1C shows localization of these residues in zfP2X4 structure. The V49, Y54 and Q55 residues proximal to the TM1 are situated at the bottom of the lateral ion access portal. In contrast, the F324 and G325 residues are found at the top segment of the lateral fenestration, at the level of conjunction between the central and extracellular vestibule. In further studies, we focused on these five residues.

### Role of V49 Residue in Receptor Trafficking

**Figure 2:**
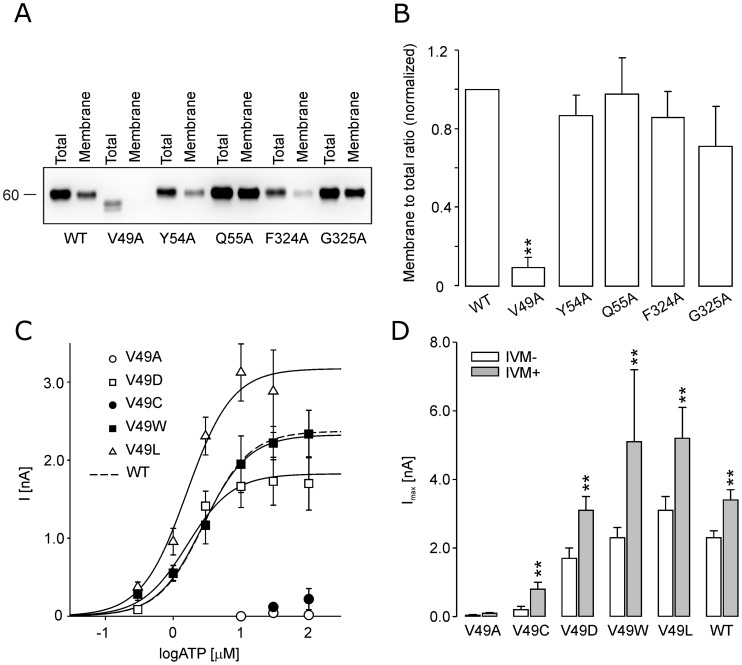
The membrane expression of the low-responsive alanine mutants and functional characterization of the V49-rP2X4R mutants. (A) Western blots showing the expression pattern of the rP2X4R-WT and V49A, Y54A, Q55A, F324A and G325A mutants. (B) Densitometry quantification of the membrane/total ratio for five low-active mutants. The data are expressed as the mean ± SEM of 4 Western blot images, **p<0.01 between WT and alanine mutants. (C) The ATP concentration response curves of the WT and V49 mutant receptors. All concentration-response curves shown in this and following figures were generated using Hill coefficient of 1.3 obtained for WT receptor (dashed line). (D) The augmentation of the maximum current amplitude of the WT and V49 mutants by ivermectin (IVM). The I_max_ was determined prior to the IVM application (white bars) and 2–6 min after application of 3 µM IVM (gray bars). The mean ± SEM from 5–18 cells per mutant is shown; **p<0.01 between control (−IVM) and ivermectin-treated (+IVM) receptors.

The unique position of V49, Y54, Q55, F324 and G325 residues in the rP2X4R could indicate their relevance in gating but could also reflect altered trafficking of mutant receptors. To test the second hypothesis, we performed quantitative western blot analysis using membrane fractions of total protein derived from HEK293T cells expressing alanine mutant and WT receptors. The V49A mutant showed low or almost no expression in the plasma membrane fraction, whereas other mutants were present in variable quantities (Fig. 2A and B). Thus, for the V49A mutant, the loss of receptor function reflects the lack of its expression at the plasma membrane. In contrast to the V49A and V49C mutants, substitution of the valine at position 49 with another non-polar (V49L, V49W) residue or a negatively charged residue of similar size (V49D) did not significantly alter the receptor function compared with WT (Fig. 2C and Table 1). Furthermore, 2–6 min of preincubation with 3 µM IVM augmented the I_max_ values for these mutants 1.7- to 2.2-fold, that was comparable with 1.5-fold augmentation observed in cells expressing the WT receptor (Fig. 2D). Together, these results indicate that trafficking but not gating of the rP2X4R depends on the V49 residue.

### Involvement of Y54 and Q55 Residues in Channel Gating

**Figure 3:**
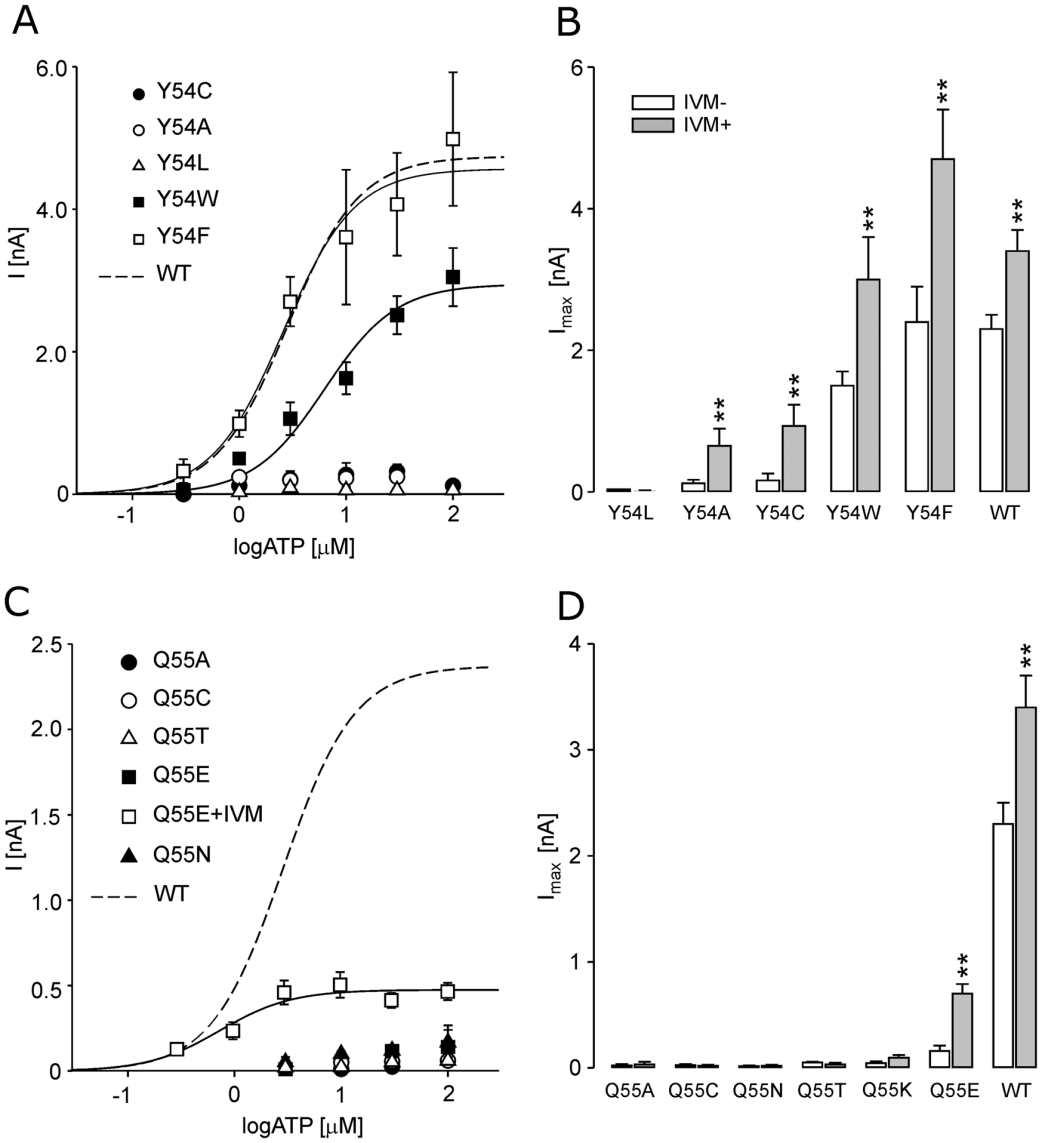
The effects of the Y54 and Q55 mutations on rP2X4R function. (A) The ATP-concentration dependence curves for the WT and Y54 substitution mutants. (B) The I_max_ values for the WT and Y54 substitution mutants in the presence (gray bars) and absence (white bars) of IVM. (C) The ATP concentration response curve for the Q55 mutants. (D) The I_max_ values of the Q55 mutants measured in the absence (white bars) and in the presence (gray bars) of IVM. The data are shown as the mean ± SEM values from 5–26 cells per mutant; **p<0.01 between control (−IVM) and ivermectin-treated (+IVM) receptors.

The Y54 alanine and cysteine mutants exhibited very low (I_max_ <0.2 nA) activity (Table 1 and Fig. 3A). Contrary to the WT and V49A-P2X4R mutant, the current amplitude of Y54A and Y54C mutants was augmented by IVM 5.4- and 5.8-fold, respectively (Fig. 3B). These results indicate that substitution of Y54 with alanine and cysteine led to a rightward shift in the potency of ATP for mutants, and that the functionality of these receptors was partially restored by IVM, which increases the frequency of channel openings [22] and sensitizes P2X4R to ATP [19]. The activity of rP2X4R was fully rescued by introducing phenylalanine and tryptophan, but not leucine (I_max_ <0.1 nA), mutations to Y54 (Table 1 and Fig. 3A and B), further indicating that an aromatic residue at position 54 is essential for the receptor function.

The Q55A and Q55C mutants were non-functional and IVM treatment was ineffective (Fig. 3C and D and Table 1). Introducing amino acids of similar structure at Q55 (Q55N, Q55E, Q55T and Q55K) did not rescue the receptor function (Fig. 3C). The function of Q55E was partially restored by the treatment with IVM (4.4-fold augmentation), whereas IVM was ineffective with other mutants (Fig. 3D). Thus, it is reasonable to conclude that substitutions of conserved residues Y54 and Q55 above TM1 with alanine or cysteine cause a stronger rightward shift in the potency of ATP for P2X4R than mutation of V49-P2X4R residue.

### Characterization of the F324 and G325 Mutants

**Figure 4:**
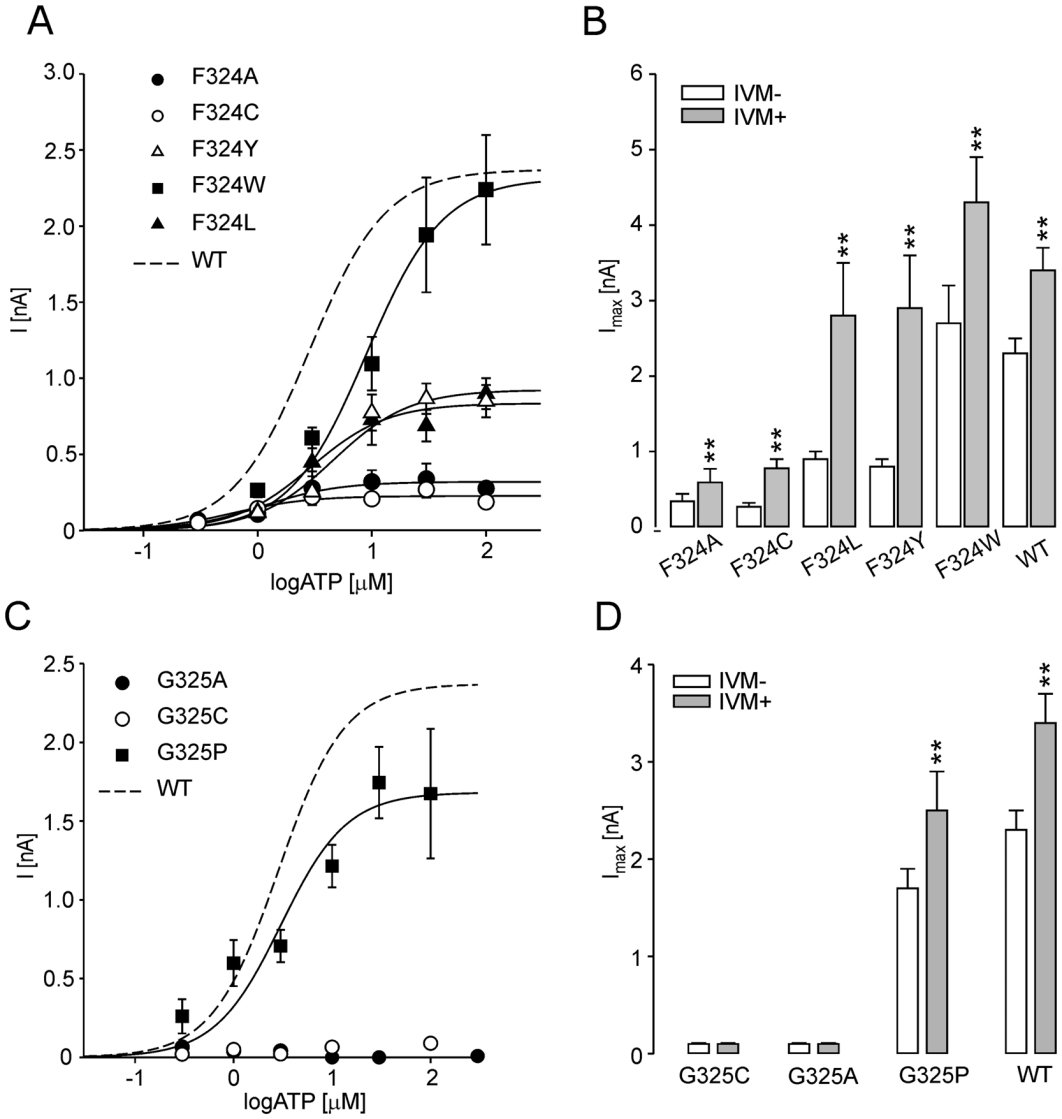
The effects of the F324 and G325 mutations on rP2X4R function. (A) The ATP-concentration response curves for the single point mutants at position F324. (B) The I_max_ values of the F324 mutants measured in the absence (white bars) and presence (gray bars) of IVM. (C) The concentration-response curve for the rP2X4-G325P (symbols) and WT receptor (dotted line). (D) The I_max_ values of G325 mutants measured in the absence (white bars) and in the presence (gray bars) of IVM. The mean ± SEM values from 7–23 cells per mutant are shown; **p<0.01 between IVM-untreated (-IVM) and -treated (+IVM) receptors.

**Figure 5:**
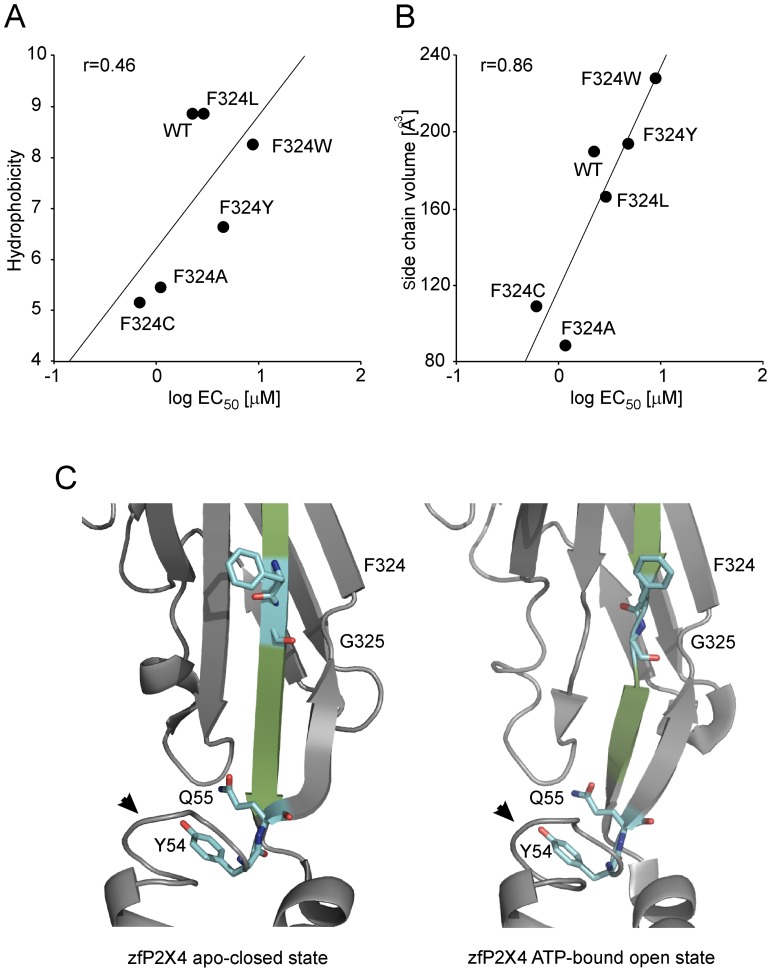
The effect of hydrophobicity and the size at position 324 on ATP potency and the localization of the F324 and G325 residues in the rP2X4R molecule. (A and B) The correlation between the EC_50_ values with the hydrophobic effect (A) and the change in side chain residue volume (B). (C) Both the F324 and G325 residues are within the β-sheet (in green) connecting the ATP binding site and the pore in the zfP2X4 apo-closed state (left) and outside the β-sheet in the ATP-bound open state (right); rP2X4 numbering. Notice the stable position of Y54 and Q55, and conserved protein fold above TM1 (arrowhead) both in the closed and open state.

The F324A and F324C mutants exhibited significantly reduced currents (I_max_ <0.4 nA; Table 2 and Fig. 4A), and preincubation with IVM increased the current amplitude by 1.7- and 2.9-fold, respectively (Fig. 4B), comparable to that observed with the WT receptor. The EC_50_ value and time course of F324A current were also similar to the WT receptor (Fig. 1B and Table 2). The mutation of F324 to another non-polar (F324L, F32W) residue or a polar aromatic (F324Y) residue partially or fully rescued the receptor function (Table 2, Fig. 4A and B). For the F324 mutants, there was linear correlation between the log EC_50_ values and the hydrophobicity (r = 0.46; Fig. 5A) and the volume (r = 0.86; Fig. 5B) of the amino acid side chain substituent. These data suggest that hydrophobicity and the size, rather than aromaticity, at position 324 are important for the proper receptor function.

The G325A and G325C mutants were not active in the absence and presence of IVM (Figs. 4C and D). The G325P mutant had a EC_50_ and I_max_ value comparable to the WT receptor (Fig. 4C). Furthermore, this mutant showed a comparable degree of IVM-induced I_max_ augmentation to the WT receptor (Fig. 4D). The G325 and F324 residues are located in the breakage point of the β-sheet in P2X4R open state (Fig. 5C). These data indicate that when the receptor undergoes activation-induced conformational changes, the G325 could operate as a hinge.

## Discussion

Through alanine- and cysteine-scanning mutagenesis of two sequences that contribute to formation of extracellular vestibules and lateral fenestrations, we show that a majority (53%) of the individual substitutions of residues V47–V61 and F324–N338 with alanine and cysteine did not significantly affect the function of rP2X4R. Among them, the F48, K52, K326, G328, I332, I333 and T335 residues are well conserved among the mammalian subunits. The replacement of another eight residues (V47, E51, D58, A327, F330, E334, I337 and N338) by alanine and/or cysteine significantly, but not critically, affected the receptor’s EC_50_ and I_max_ values. Among the mammalian subunits, these residues are relatively well conserved: V47 in five, E/D51 in four, D/E58 in six, A327 in six, F330 in seven, P334 in six, I337 in five and N338 in three. Previous studies have shown a role for V47, I337 and N338 residues in P2X2R gating and pore formation [41], [42], [38], [43], [44]. In P2X1R, the A327 amino acid residue may also contribute to channel gating [39]. Charged residues E56 and D58 are important for forming an access pathway for the ion entrance [29] and residue E51 contributes to the high Ca^2+^ permeability of P2X4R [45], [46], [28]. Further studies are needed to characterize the potential role of F330 and P334 residues in the receptor’s function.

Here we identified five amino acid residues that are critical for rP2X4R function: V49, Y54, Q55, F324 and G325. These residues are also present in zfP2X4R and among the mammalian P2XRs, Y54, Q55 and G325 are fully conserved, V49 is present in five receptors, and F324 is non-conserved across P2X subtypes. In general, the loss of receptor function by substituting these residues with alanine and cysteine could reflect the altered trafficking of mutants to the plasma membrane or the loss of responsiveness to ATP, whereas their possible participation in ATP binding is highly unlikely in the light of recent crystallization of zfP2X4R with ATP bound [26].

However, here we show that trafficking of receptors was significantly affected by mutation of V49 but not other residues. The V49A was sensitive to IVM similarly as the WT-P2X4R, further arguing that gating of a small population of receptors that reached the plasma membrane was not affected. In addition, the V49D mutant is fully functional, although repulsive forces may form between the aspartate carboxyl side chain and the negatively charged phospholipids. Similarly, the V49W mutation did not alter the receptor’s function [47], although bulky amino acids could accumulate in this part of the helix (F48–W49–W50). The V50-P2X2R mutant is also functional [38], [43]. Thus, the V49 residue may play a role in trafficking of the rP2X4R, rather than destabilizing membrane anchoring inside membrane or influencing ion gating.

Consistent with our data, previous studies performed on P2X2R also showed that the Y54C and Q55C mutants (rP2X4 numbering) did not form functional channels [42], [31], but both studies make no further assessment of these nonfunctional mutants. Cysteine-scanning mutagenesis of residues E52–G96 in hP2X1R showed that all extracellular vestibule mutants are functional; therefore, there are subtype differences in the ability of the receptor to tolerate Q55 and Y54 mutations [32], [34]. We report here that that other polar or charged mutations at Q55 position were not able to rescue the receptor function, while the Y54F and Y54W mutants responded to ATP, indicating the relevance of aromatic residue at the second position. It is surprising that the Q55N mutant was entirely nonfunctional in spite of significant structural similarity between the Gln and Asn side chains. The Y54A/C-P2X4R function is partially rescued by IVM as well as the Q55E-P2X4R function, whereas Q55A, Q55C, Q55N Q55T, and Q55K mutants were IVM insensitive. Because IVM causes a leftward shift in the sensitivity of receptors to ATP [19], it is reasonable to suggest that the Y54 residue contributes to gating. We may speculate that this is also the case for Q55 residue, but that the loss of responsiveness to ATP is more severe and could not be rescue by IVM other than for Q55E.

**Figure 6:**
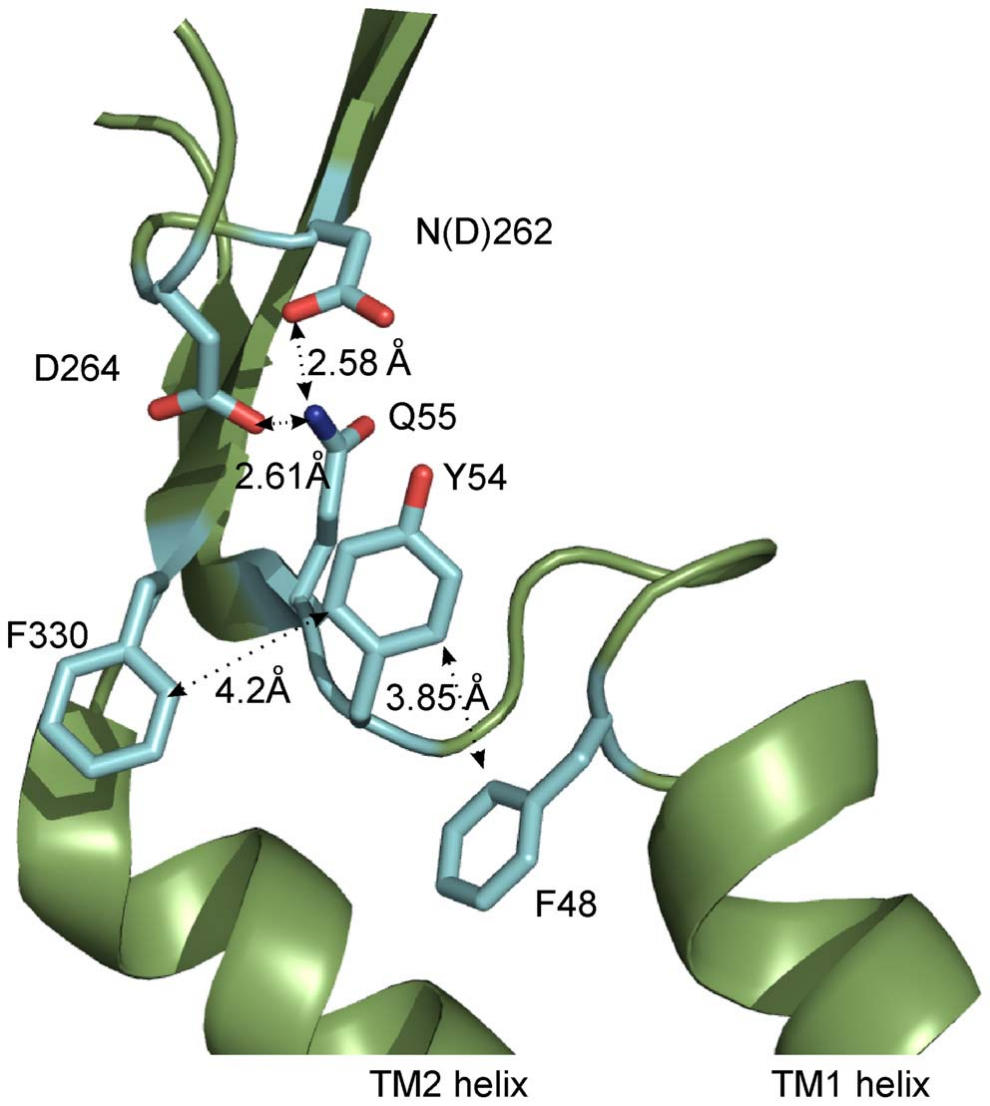
The possible interactions of Y54 and Q55 with other residues in the same P2X4R subunit. Residue Y54 can form stacking interactions with F330 or F48, Q55 can form H-bonds with N262 (zfP2X4 has aspartic acid in corresponding position) or D264. The structure shows the zfP2X4R in the apo-closed state, numbering is rP2X4R.

A recently published crystal structure of zfP2X4R [26] showed that the Q55 residue forms hydrogen bonds with the N262 (zfP2X4 has aspartic acid in corresponding position) or D264 residues from the same subunit (Fig. 6). Interestingly, an unchanging structural fold is present within 7Å of the Q55 and Y54 residues when the channel is either open or closed [26]. Our results indicate that even the slightest change in the size and geometry of Q55 residue disrupts this conserved peptide fold and disrupts receptor function. Therefore, we conclude that the structure fold proximal to the Q55 residue must be maintained for the channel to be functional in either conformational state.

In a study addressing the molecular dynamics simulations of the P2X4R, the Y54 residue was also hypothesized to form a hydrogen bond with the D264 residue, implying that this interaction is important for channel opening [48]. Our results suggest that, if such interaction exists, it is not functionally important because while Y54F mutant lacks a hydroxyl group, it forms a functional receptor. Our study further shows that stacking interactions are most likely important for the functionality of the Y54 mutants because an aromatic residue is crucial at this position for receptor function. The possible interaction partners of Y54 are F48 from TM1 and F330 from the TM2 of the same subunit (Fig. 6). In agreement with this hypothesis, we found here that the F330C mutant exhibited a significantly reduced I_max_ current (Fig. 1) and previously we have shown that numerous aromatic residues in the upper part of TM1 are important for P2X4R function [49]. Therefore, we conclude that Q55 and Y54 could play a crucial role in the mutual axial orientation of the TM1 and TM2 helices to a position that is functionally important for channel gating.

We also identified the G325 residue of rP2X4R to be functionally important because both G325A and G325C were not responsive to ATP. The rescue effect of G325P mutant clearly shows the importance of this region for vestibule enlargement, because proline substitution makes an angular disruption and brakeage of β-sheet [50] and the topology of G325 in the open and closed state of zfP2X4R reveals that the β-sheet above TM2, connecting ATP binding domain with the channel pore, is disrupted in the position of G325 (Fig. 5C). These results indicate that angular brakeage of this region is a prerequisite for open-closed state transition and that G325 could be a flexible hinge, which might be crucial for the twisting of the β-sheets and extracellular vestibule widening after receptor activation.

Although the G325 residue is conserved along the P2XR isoforms, its critical functional importance is shown only in rP2X4R. In the P2X1R, the G321C mutant (equivalent to G325C in P2X4) was normal [51] but exhibited a modified response to ATP in the presence of MTS reagents [39]. Other study with rP2X2 also showed that H319C and G320C receptor mutants (analogous to F324C and G325C) are functional [31], [44]. In our study, cysteine and alanine mutants of F324 residue had reduced I_max_, which was found also by others [52], but were partially rescued by introducing F324L, F324Y, and F324W mutations. The correlation between the EC_50_ value and hydrophobicity for a particular F324 substituent indicates that the size of the residue and the ability to form hydrophobic interactions are important for receptor function. This intersubunit interaction could stabilize the closed state. Both the F324 and G325 residues are located in a non-structuralized region if the β-sheet proximal to the TM2 is disrupted [26]. Therefore, the F324 mutation may obstruct vestibule enlargement.

In conclusion, we identified four amino acid residues that are critically important for rP2X4R function, although their trafficking was not affected. Residues Q55 and Y54, together with interaction residues, establish a fixed protein fold above TM1, both in the open and closed states, which is important for gating functions. The residue G325 probably operates as a hinge during conformational changes that occur upon activation and together with F324 is important for the proper three-dimensional structure of P2X4R vestibule and vestibule widening upon ATP binding. The V49 residue is proven to be important for receptor trafficking. These results contribute in favor of the isoform specific, multiple functions of the amino acid residues comprising the extracellular vestibule in P2XR.
